# Microalgal photophysiology and macronutrient distribution in summer sea ice in the Amundsen and Ross Seas, Antarctica

**DOI:** 10.1371/journal.pone.0195587

**Published:** 2018-04-10

**Authors:** Anders Torstensson, Agneta Fransson, Kim Currie, Angela Wulff, Melissa Chierici

**Affiliations:** 1 Department of Biological and Environmental Sciences, University of Gothenburg, Göteborg, Sweden; 2 Norwegian Polar Institute, Fram Centre, Tromsø, Norway; 3 Department of Earth Sciences, University of Gothenburg, Göteborg, Sweden; 4 National Institute for Water and Atmospheric Research Ltd (NIWA) / University of Otago Research Centre for Oceanography, University of Otago, Dunedin, New Zealand; 5 Department of Marine Sciences, University of Gothenburg, Göteborg, Sweden; 6 Institute of Marine Research, Tromsø, Norway; University of Tasmania, AUSTRALIA

## Abstract

Our study addresses how environmental variables, such as macronutrients concentrations, snow cover, carbonate chemistry and salinity affect the photophysiology and biomass of Antarctic sea-ice algae. We have measured vertical profiles of inorganic macronutrients (phosphate, nitrite + nitrate and silicic acid) in summer sea ice and photophysiology of ice algal assemblages in the poorly studied Amundsen and Ross Seas sectors of the Southern Ocean. Brine-scaled bacterial abundance, chl *a* and macronutrient concentrations were often high in the ice and positively correlated with each other. Analysis of photosystem II rapid light curves showed that microalgal cells in samples with high phosphate and nitrite + nitrate concentrations had reduced maximum relative electron transport rate and photosynthetic efficiency. We also observed strong couplings of PSII parameters to snow depth, ice thickness and brine salinity, which highlights a wide range of photoacclimation in Antarctic pack-ice algae. It is likely that the pack ice was in a post-bloom situation during the late sea-ice season, with low photosynthetic efficiency and a high degree of nutrient accumulation occurring in the ice. In order to predict how key biogeochemical processes are affected by future changes in sea ice cover, such as *in situ* photosynthesis and nutrient cycling, we need to understand how physicochemical properties of sea ice affect the microbial community. Our results support existing hypothesis about sea-ice algal photophysiology, and provide additional observations on high nutrient concentrations in sea ice that could influence the planktonic communities as the ice is retreating.

## Introduction

Sea ice plays an important role in Antarctic marine biogeochemical cycles, both in terms of physical, chemical and biological processes [1–4]. Algae, in particular diatoms, inhabiting the brine channels of sea ice, contribute to the primary production in ice covered polar oceans and provide an important food source to higher trophic levels [5–7]. As ice algal biomass generally peaks before the pelagic bloom near the sea-ice edge, the ice algal community can act as an important seeding population to the pelagic bloom, if dispersed into favorable conditions in the marginal ice zone [4, 8, 9]. In addition, organic matter derived from algae plays an important role in sea-ice microbial ecology. Strong bacteria-algae interactions have been reported in many other marine systems, and it has been suggested that the microbial loop is important for recycling of organic matter in sea ice [10–12]. Sea ice also acts as a source of organic and inorganic nutrients for the planktonic community as the ice is retreating [13]. However, nutrient dynamics in pack ice have been identified as overlooked, and relatively little data is available from the Amundsen Sea sector [2].

Sea ice is generally characterized as a habitat with steep gradients in temperature, salinity, radiation and nutrient concentrations [14]. However, during the Austral summer, the Southern Ocean is dominated by relatively warm, surface-flooded sea ice that does not follow the typical *in situ* bulk salinity profile (C-shaped) observed in young first-year ice in the Arctic [15]. Instead, salinity profiles are often isohaline or follow a S-shaped form due to snow loading and ice melting [15, 16]. As first-year sea ice warms and reaches a critical brine volume of 5%, the ice is generally considered permeable according to percolation theory [17], which facilitates nutrient replenishment in the sea ice. These properties create complex microenvironments, and facilitate the establishment of a productive sympagic microbial ecosystem that is not challenged by sinking below the euphotic zone. Microorganisms are commonly found in high densities throughout the vertical column of pack ice [4, 18, 19]. Although pack ice is the dominating ice type in the Southern Ocean, most studies of sea-ice algal physiology have been performed on bottom-ice communities in land fast ice close to the continent [20–24]. Due to the reduced amount of surface flooding, land-fast ice and Arctic sea ice are in general characterized by a dense band of algae concentrated at the bottom 0.1 m of the ice, near the ice-water interface. In contrast, the microalgae present in Antarctic pack ice are distributed throughout the ice column, which makes it difficult to predict the biomass maximum and the site of highest microbial activity. In turn, this also complicates attempts to model primary production and other biogeochemical processes in sea ice [25, 26], which will become increasingly important as sea-ice volume and extent are decreasing in some areas of the Southern Ocean [27]. In order to understand these dynamics, identification of key environmental drivers affecting the biomass distribution and photophysiology of pack-ice algae is necessary.

Sea-ice algae are well adapted to low-light conditions in order to grow in the shaded environment of snow and ice [14, 28]. However, a previous study on kinetics of photosystem II (PSII) has shown that pack-ice communities display a rather plastic response to elevated radiation intensities, and can withstand relatively high intensities of photosynthetic active radiation (PAR) [29]. Sea-ice temperature, brine salinity and light levels often co-vary through the vertical profile of sea ice [14]. Therefore, separating the individual drivers (such as radiation and salinity) of microalgal physiology across ice types becomes complicated. A number of laboratory studies have been performed to address responses to light, salinity, temperature and CO_2_ concentrations in algal cultures [28, 30–32]. However, as natural sea-ice assemblages can have a high diversity and complex community structure, results from culture experiments are generally very limited in terms of ecological scaling. Due to logistical reasons, many sea-ice covered areas in the Southern Ocean are also under-sampled, both with respect to microalgal biomass and photophysiology.

It has previously been suggested that the photophysiology of sea-ice algae in the Amundsen Sea is primarily controlled by radiation and nutrient availability [33]. Snow cover significantly increases the attenuation of light in the ice, and indirectly affects microalgal physiology [33]. Although salinity gradients are less distinct in summer Southern Ocean pack ice, a recent study in this area suggests that salinity is in fact a major driver of microbial diversity in this ice-type [34], and could have important consequences for microbial acclimation. Hence, the microenvironment of sea ice is highly variable and plays an important role in the ecophysiology of psychrophilic microorganisms. In the present study, we have assessed photophysiology and macronutrient distribution in sea-ice samples collected from 14 sites in the Amundsen and Ross Seas regions of Antarctica. In total, 236 high-resolution sea-ice core sections (0.1 m) were analyzed using pulse amplitude modulation (PAM) fluorometry from a transect in a relatively under-sampled sector of the Southern Ocean. PAM fluorometers can be used to non-invasively measure quantum yields of photosystem II (PSII), quenching coefficients and to estimate the rate of the electron transport chain in the thylakoid membranes, and are often used to study stress and light adaptation in plant and algae sciences [35–38].

## Materials and methods

### Study site

**Fig 1:**
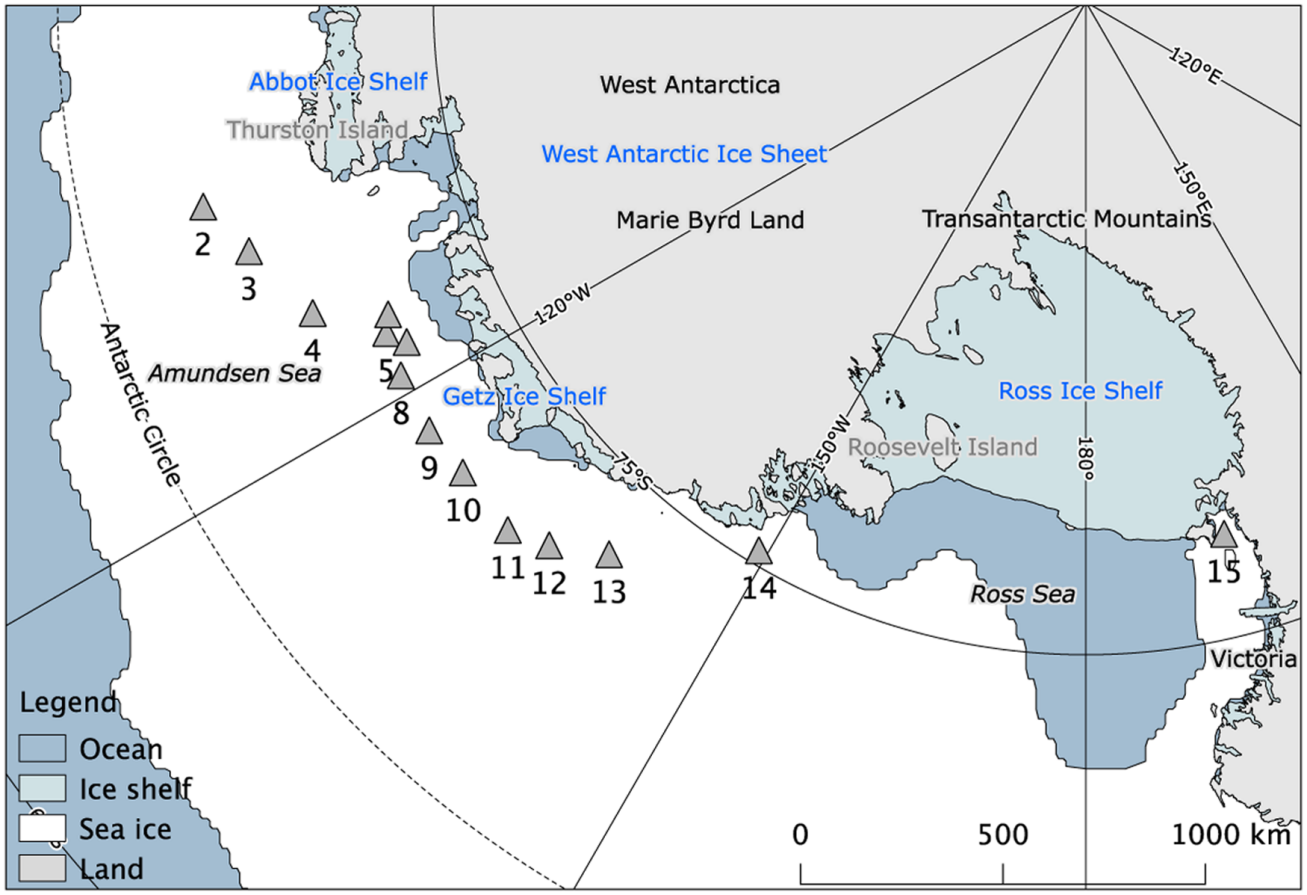
Sea-ice sampling stations during the Oden Southern Ocean 2010/2011 cruise. Data of average sea extent from December 2010 was provided by the National Snow and Ice Data Center (http://nsidc.org/). Map was created using the Quantarctica 2.14 QGIS-package, developed by the Norwegian Polar Institute (www.quantarctica.org).

Sea-ice samples were collected from 14 sites (labeled 2–15 according to Torstensson [34]) in the Amundsen and Ross Seas, Antarctica, during the Oden Southern Ocean cruise from December 2010 to January 2011 (Table 1, Fig 1). The Swedish Polar Research Secretariat granted permission for the fieldwork. Separate sea-ice cores for biological (variable fluorescence, photosynthetic pigments and bacterial abundance) and physicochemical (temperature, salinity, inorganic macronutrients and carbonate chemistry) analyses were collected within a 1 m radius, from sampling sites with homogenously thick sea ice [34]. In total, 23 ice cores were sampled for biological parameters using a 0.12 m-diameter ice corer. Duplicate cores from each site were sampled when logistically possible, which resulted in duplicates from most stations (Table 1). Under-ice water was sampled approximately 1 m below the ice water using a pump attached to an L-arm. Surface slush layers were not sampled in the present study. Sea-ice temperature was recorded immediately after recovery of physicochemical cores using a digital thermistor (Ama-digit ad 15 th, Amarell GmbH & Co, Kreuzwertheim, Germany) with an accuracy of ±0.1°C. The cores for analyses of biological parameters were immediately covered in opaque black plastic bags for protection against direct sunlight. All cores were cut into 0.1 m sections and placed in gas-tight Tedlar^®^ bags and transported to the ship in a dark and insulated box. The sections from the physicochemical cores were vacuum-sealed and left to thaw overnight. Salinity in the melted sea-ice sections (bulk salinity) was measured using a conductivity meter (Cond 310i, WTW GmbH, Weilheim, Germany) with a resolution and accuracy of ±0.05 units. Brine salinity and volume were calculated using sea-ice temperature and bulk salinity [39, 40]. The biological sections were crushed and mixed with a small amount (60 ml) of 0.2 μm filtered seawater (~0°C) to quickly extract sea-ice algae from the ice matrix for measurement of chl *a* fluorescence. This rapid extraction of cells resulted in a final salinity of > 28, which is recommended for minimizing the stress during physiological measurements of sea-ice algae, such as chl *a* fluorescence [41]. The subsample used for chl *a* fluorescence was returned and pooled with the crushed core-sections after measurement. To minimize osmotic and thermal stress for the quantitative biological analyses (photosynthetic pigments and bacterial abundance), the ice-water mix was thawed in darkness for 12–20h together with 1 L, 0.2 μm filtered and pre-chilled seawater (approximately 1:1 ice:water ratio). Samples were processed immediately after melting.

**Table 1:** Station list, environmental and biological characteristics of the sea ice. Stations are numbered according to a previous study [34]. Bacterial abundance and chl *a* concentrations are depth-integrated throughout the sea ice column, and presented in either single or duplicated cores.

Station	*n*	Date	Latitude (°S)	Longitude (°W)	Ice thickness (m)	Snow depth (m)	Bacteria (10^11^ cells m^−2^)	Chl *a* (mg m^−2^)
2	1	17-Dec-2010	69.28	103.0	0.80	0.20	5.4	2.2
3	1	18-Dec-2010	70.01	106.6	0.69	0.30	7.7	2.0
4	1	19-Dec-2010	70.91	111.9	1.49	0.12	10.3	3.7
5	2	20-Dec-2010	72.25	115.3	0.94	0.35	3.5, 4.1	1.3, 0.8
6	2	21-Dec-2010	72.46	114.1	1.04	0.32	1.8, 1.9	2.2, 2.7
7	1	26-Dec-2010	72.57	116.6	2.32	0.43	13.1	6.7
8	2	27-Dec-2010	72.11	118.6	1.49	0.55	3.2, 5.0	3.2, 4.4
9	2	29-Dec-2010	72.03	123.1	1.19	0.25	12.7, 13.2	18.8, 25.9
10	1	30-Dec-2010	72.10	127.1	1.92	0.25	4.8	4.4
11	2	2-Jan-2011	72.05	132.4	0.93	0.10	9.5, 19.6	0.7, 7.3
12	2	2-Jan-2011	72.48	135.4	1.32	0.17	6.1, 9.1	2.2, 3.2
13	2	4-Jan-2011	73.26	139.2	0.85	0.07	2.7, 5.1	0.5, 0.6
14	2	6-Jan-2011	75.33	149.2	1.40	0.38	4.6, 4.8	2.6, 3.3
15	2	10-Jan-2011	77.35	E 165.4	1.56	0	1.1, 1.3	0.2, 0.2

### Variable fluorescence

The maximum and effective quantum yield of photosystem II (F_v_/F_m_ and ΔF/F_m_’, respectively) were measured upon return to the ship by pulse amplitude modulation (PAM) fluorometry, using a Water-PAM equipped with blue light emitting diodes (Walz Mess- und Reigeltechnik, Effeltrich, Germany). The emitter-detector was kept cold (~0°C) during all measurements, and all samples were stored dark and cold until processed and analyzed. All samples were analyzed within 1–4 hours from collection in the field, and all sample processing was performed in a dark laboratory. Processing time was only long enough to increase the light saturation point (E_k_) in two of the eleven sampled stations (S1 Fig), and thereby considered negligible in the overall study. Samples were dark adapted at ~0°C for a minimum of 15 min before any measurement. Dark incubation times were verified to be sufficient by multiple F_v_/F_m_ measurements over two hours. After dark adaptation, minimum fluorescence (F_0_) was determined by applying a low level of measuring light and the maximum fluorescence (F_m_) by exposing the sample to a short pulse of saturation light (> 1000 μmol photons m^−2^ s^−1^ for 0.8 s). F_m_’ was determined for light-exposed cells during rapid light curves (RLCs) and non-photochemical quenching (NPQ) analysis. Variable fluorescence (F_v_ = F_m_−F_0_) and maximum quantum yield of PSII (F_v_/F_m_) for each sample were determined in duplicated measurements for improved accuracy. RLCs were performed by measurement of ΔF/F_m_’ ((F_m_’−F_0_) / F_m_’) of quasi-adapted (15 s) cells at nine levels of actinic light (0, 13, 20, 30, 46, 69, 102, 154 and 228 μmol photons m^−2^ s^−1^, as calibrated with a WALZ Micro Quantum Sensor US-SQS/IB) in the emitter-detector unit. Relative electron transport rate (rETR) was calculated by ΔF/F_m_’ multiplied by PAR intensity. Photosynthetic parameters (rETR_max_, E_k_, and α_PSII_) were calculated according to Jassby and Platt [42], fitted by the Nelder-Mead method in the R package phytotools [43, 44]. NPQ was determined in a separate sample after exposure to 66 μmol photons m^−2^ s^−1^ for 270 s, and calculated by (F_m_−F_m_’)/F_m_’. Incubation time was regularly verified to be saturating for quenching parameters by light-inductive curves. Due to time constraints, NPQ was not measured at every station.

### Photosynthetic pigments

Directly after thawing, 95–260 ml of melted sea ice was filtered on 25 mm GF/F filters. The filters were immediately flash-frozen in liquid nitrogen and stored at −80°C until extraction. Filters were extracted in 2 ml acetone/methanol (80:20) and sonicated using a Vibra-cell sonicating probe, operating at 80% in 5-s pulses. High performance liquid chromatographic (HPLC) analyses of the extracts were performed [45], using an absorbance diode array-based detector (Spectraphysics UV6000LP). A 150 × 3.0 mm Phenomenex Kinetex 2.6-μ C18 100A column was used for separation. Pigments were identified by their retention time and absorbance spectra (400–700 nm) and compared to pigment calibration standards, provided by DHI Water and Environment, Denmark. Concentrations of the main photosynthetic pigments, chl *a* and fucoxanthin, were either integrated over area or corrected to brine volume according to Frankenstein and Garner [40].

### Bacterial abundance

Melted sea-ice samples were fixed in 1% glutaraldehyde (final concentration) and stored at −80°C until analysis. Bacteria were stained with SYBR Green Nucleic Acid Gel Stain (Invitrogen, final dilution 5 × 10^−5^) for 10 min in darkness. Cell counting was performed with a FACScalibur flow cytometer (BD Biosciences, Mountain View, USA), using side scattering light (SSC) and green fluorescence (FL1) for detection. Flow rate was determined with an internal standard of 1 μm FluoSpheres (Invitrogen, Eugene, OR, USA). The concentration of FluoSpheres was quantified by triplicated measurement together with BD Trucount absolute counting beads (BD Biosciences, Mountain View, USA). Bacterial abundances were either integrated over area, or corrected to brine volume according to Frankenstein and Garner [40].

### Dissolved inorganic carbonate chemistry

Total alkalinity (A_T_) of melted sea ice was determined by potentiometric titration of 40 mL sample in open cell with 0.05 mol l^−1^ hydrochloric acid using a Titrino system (Metrohm, Switzerland) [46]. The precision was ±2 *μ*mol kg^−1^, obtained by triplicate analysis of one sample on a daily basis. The accuracy was checked against a certified reference material (CRM) supplied by Andrew Dickson (Scripps Institution of Oceanography, San Diego, USA) measured at the beginning and at the end of 20 samples.

pH was determined spectrophotometrically, using m-cresol purple and a diode-array spectrophotometer, HP8452 [47]. The analytical precision was estimated to ±0.002 pH units, which was determined by triplicate analysis of one sample every day. The pH of the indicator solution was measured daily using a 0.2-mm flow cell, this was then used as correction for the perturbation caused by the addition of the indicator solution [48].

Total dissolved inorganic carbon (DIC) was calculated based on measured data of pH and total alkalinity (A_T_), salinity and temperature, using the carbonate speciation program CO2SYS 2.1 [49]. We used the carbonate dissociation constants (K_1_ and K_2_) of Mehrbach [50] as refitted by Dickson and Millero [51], and the KSO_4_ determined by Dickson [52]. Brine-corrected A_T_ and DIC (derived from A_T_ and pH) were used to recalculate the carbonate system in the brine.

### Inorganic macronutrients

Melted samples from the physicochemical cores were 0.2 μm filtered and stored at −80°C until analysis of dissolved inorganic macronutrient concentrations (nitrite + nitrate, phosphate and silicic acid). Samples were analyzed at the Sven Lovén Center for Marine Infrastructure, Kristineberg, Sweden, using colorimetric methods [53]. For comparison with sea ice algal photophysiology, data were normalized to brine concentration to correct for dilution during melting [40]. The conservative behaviors of the inorganic macronutrients were tested relative to bulk salinity, where theoretical dilution lines (TDL) were obtained using macronutrient concentrations in the under-ice water.

### Statistical analyses

Redundancy analysis (RDA) was performed in CANOCO 5 to explore linkages between biological and environmental variables. RDA can effectively visualize potential linear relationships of multivariate data matrices by producing an ordination plot constrained to present a combination of multiple predictor variables responsible for the majority of the total variation in a dataset. Combinations of highly correlated environmental data were excluded from the analysis, such as temperature and salinity. Bootstrapping was performed using Monte Carlo Permutation Procedure (MCPP) tests, executed with 1000 iterations.

Multiple and simple linear regressions were performed in R 3.1.1 [43], and used to describe the relationships between biomass, photophysiological and environmental parameters. Variable selection was performed by stepwise selection based on the Akaite information criterion (AIC). Simple correlations between variables were assessed with Pearson’s correlation coefficient. If necessary, data were log-transformed to compensate for normal distribution and heteroscedasticity. A probability level (p) of < 0.05 was used for statistical significance.

## Results

### Environmental parameters

All sea-ice samples in this study came from first-year pack ice (sometimes rafted ice), except for station number 15 (McMurdo Sound) where land fast ice was sampled. The sea ice in McMurdo Sound had a typical salinity and temperature profile of land fast ice, and differed significantly from the pack ice in the Amundsen Sea. Average ±SD sea-ice thickness during the entire study was 1.19 ±0.41 m, with a snow cover varying between 0–0.55 m (Table 1). The temperature in all segments varied between −0.25 and −2.65°C, which corresponds to a salinity range of approximately 5–50. Only 2.3% of the total samples had a brine volume of < 5%, and all of those samples were from the same station (station number 8). There were no significant correlations between brine salinity and ice depth, or relative ice depth, i.e. percentage of total core length (p = 0.256 and 0.559, respectively). Incoming surface PAR irradiances during the study are presented in S2 Fig.

### Biomass

**Fig 2:**
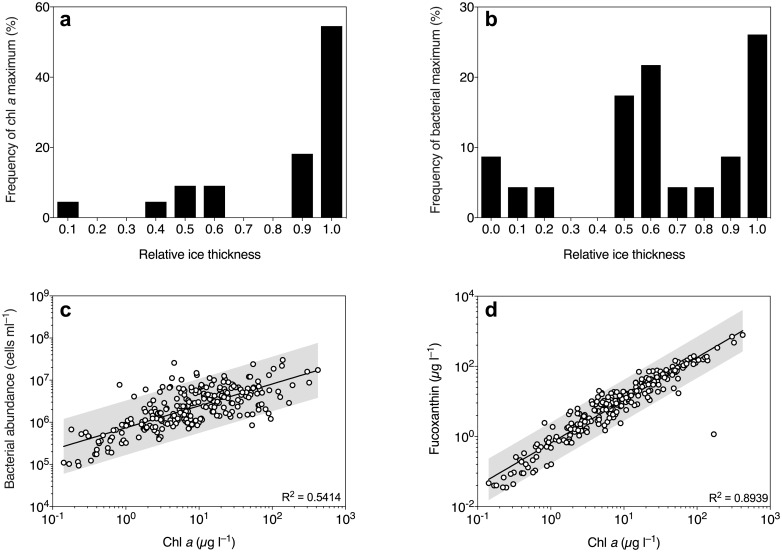
Biomass distribution in Southern Ocean sea ice during summer. Frequency distribution of the chl *a* (**a**) and bacterial abundance (b) maxima at different relative ice thicknesses (binned in 10% increments). Correlation between bacterial abundance and chl *a* concentration in the sea-ice samples (**c**). Correlation between chl *a* and fucoxanthin concentration (**d**). Concentrations are scaled to brine volume and the grey areas represent 95% prediction interval of the fitted line.

Average ±SD depth-integrated chl *a* and bacterial abundances (excluding surface slush layer) were 4.3 ±6.1 mg m^−2^ and 6.5×10^11^ ±4.7×10^11^ cells m^−2^, respectively (Table 1). Although the chl *a* maximum was generally located near the bottom section of the sea ice, it also occasionally occurred in the interior ice (Fig 2a). Bacterial concentrations ranged between 9.3×10^4^ and 3.0×10^7^ cells ml^−1^, as expressed in brine volume, and the maximum abundances were generally located either in the bottom or in the middle of the ice (Fig 2b). There was a positive correlation between bacterial abundance and chl *a* concentration (Fig 2c). Fucoxanthin was the main accessory photosynthetic pigment, and followed on average a 1.2:1.0 ratio to chl *a* concentration (Fig 2d). Although significantly correlated (p = 0.0078, S3a Fig), chl *a* concentration explained a low degree (3.1%) of the total variance in DIC. There was no significant correlation between DIC and bacterial abundance (p = 0.382, S3b Fig). The majority of samples (85%) had a brine-scaled chl *a* concentration of > 1 μg l^−1^ (Fig 2a), where the land-fast ice (station number 15) in McMurdo Sound had the lowest depth-integrated chl *a* and bacterial abundance (Table 1). Here, chl *a* was mainly observed in the bottom and top section of the ice, with average ±SD brine-scaled concentrations of 0.8 ±0.56 and 0.95 ±0.09 μg, respectively. At this station, 60% of the samples had concentrations below 0.5 μg l^−1^.

### Macronutrients

**Fig 3:**
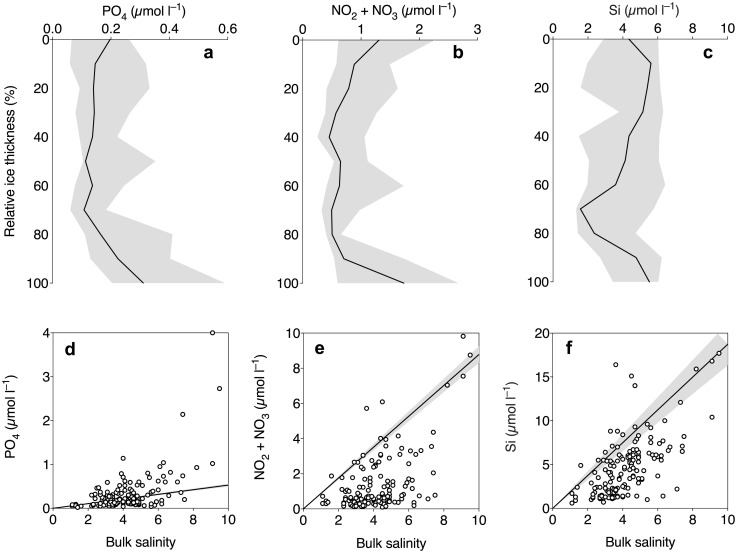
Vertical profiles (a–c) and nutrient mixing diagrams (d–f) of the inorganic macronutrients phosphate (a and d), nitrate + nitrite (b and e) and silicic acid (c and f) in Antarctic sea ice. The upper panel shows median values of bulk nutrient concentrations, plotted against the relative ice thickness (binned in 10% increments). The error bands represent the interquartile range in a–c. The lower panel shows bulk salinity plotted against bulk concentrations of macronutrients. The linear regression line in d–f represent the theoretical dilution line (±95% confidence interval), based on data from seawater collected ~1m below the sea ice.

Bulk concentrations of phosphate and nitrate + nitrite generally followed C-shaped profiles through the sea ice column (Fig 3a and 3b), whereas silicic acid was more homogenous throughout the ice (Fig 3c). Compared to the TDL, phosphate deviated both above and below the TDL (Fig 3d). Most data points of nitrate + nitrite and silicic acid concentrations were below the TDL (Fig 3e and 3f) indicating a drawdown of these macronutrients in the sea-ice samples. For nitrate + nitrite, these values were near depletion (< 0.2 μmol l^–1^) in 17 samples from middle sections of the sea ice column (Fig 3b and 3e).

**Fig 4:**
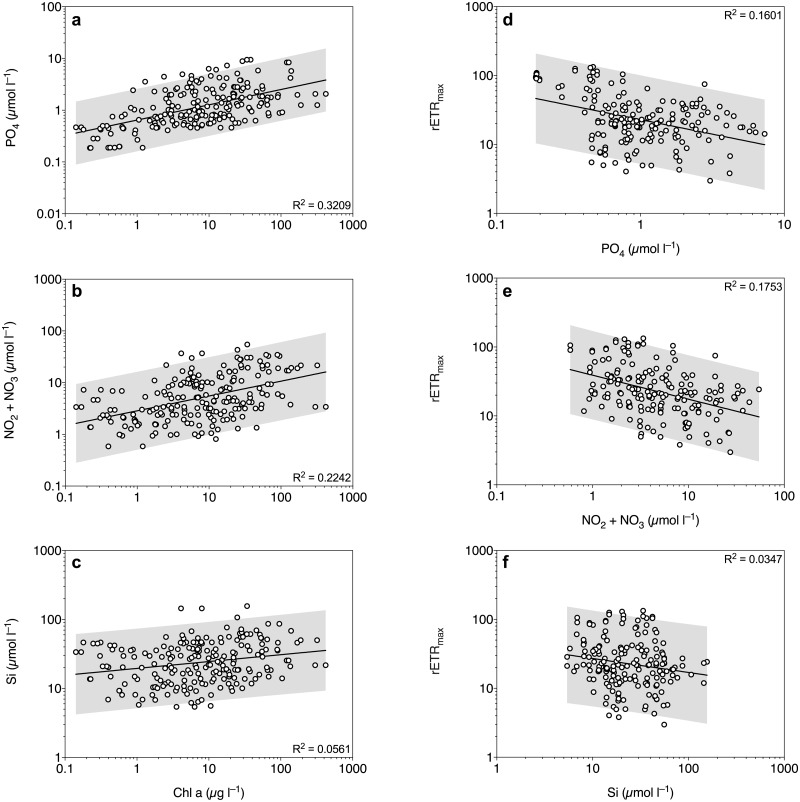
Correlation between chl *a* and inorganic macronutrient concentrations (a–c), and between PSII capacity (rETR_max_) and inorganic macronutrient concentrations (d–f). The grey areas represent 95% prediction intervals of the fitted lines. All concentrations are scaled to brine volume.

Brine-scaled Chl *a* concentrations were positively correlated with inorganic macronutrient concentrations (p < 0.0001, Fig 4a–4c). Chl *a* explained 32.1%, 22.4% and 5.6% of the variability in phosphate, nitrite + nitrate and silicic acid, respectively. Phosphate and nitrite + nitrate concentrations were also positively correlated with bacterial abundance (p < 0.0001), where bacterial abundance explained 38.9% and 12.8% of the variability, respectively. Fucoxanthin concentration was positively correlated with silicic acid concentration (p = 0.0003), but only explained 6.1% of the variability.

### Photophysiology

**Fig 5:**
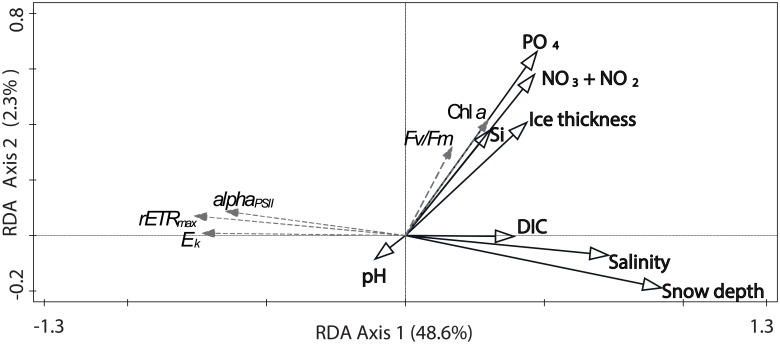
RDA ordination plot of environmental variables (black solid lines) explaining photophysiological data (grey, dashed lines). The first two RDA axes were significant (pseudo-F = 13.4, p = 0.001, 1000 permutations in Monte Carlo permutation test) and account for 51.0% of the total variation in the dataset. The environmental variables are inorganic macronutrient concentrations (nitrite + nitrate (NO_2_ + NO_3_), phosphate (PO_4_), silicic acid (Si)), brine salinity, dissolved inorganic carbon (DIC), pH, snow depth, sampling depth (ice thickness). Photophysiological data include F_v_/F_m_, rETR_max_, E_k_, α_PSII_ (alpha_PSII_) and brine-scaled chl *a* concentration.

According to the RDA, the main correlates that explained most of the variability in RLC-derived PSII performance (rETR_max_, E_k_, and α_PSII_) were snow depth, brine salinity and DIC concentration (Fig 5). The PSII parameters did not show a strong association with macronutrient levels or chl *a* concentrations (Fig 5). Instead, the RDA suggested that inorganic macronutrient concentrations were the main correlates for chl *a* distribution (Fig 5).

**Fig 6:**
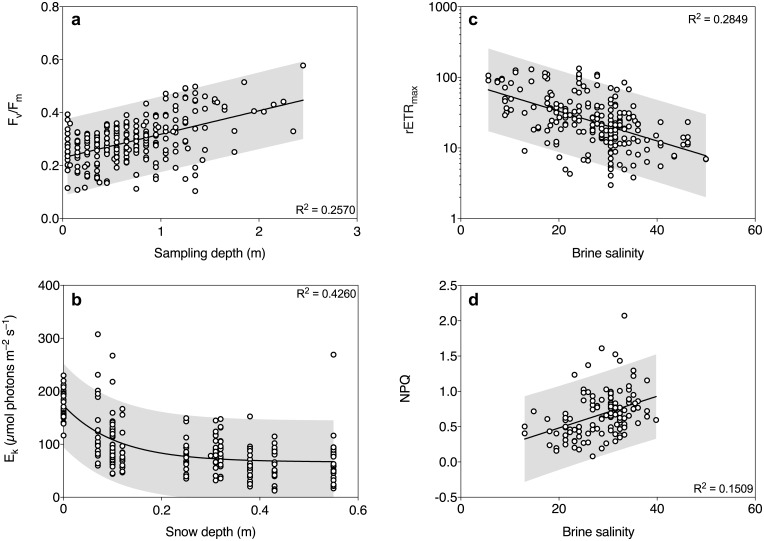
Simple linear regressions for the major drivers of PSII activity in Southern Ocean sea ice during summer. Data illustrate the relationships between sampling depth and F_v_/F_m_ (**a**), and light saturation point (E_k_) and snow depth (**b**). Maximum rate of electron transport rate (rETR_max_) (**c**) and non-photochemical quenching (NPQ) (**d**) are negatively and positively correlated with brine salinity in sea-ice algal communities, respectively. P-values are reported from Pearson’s correlation, and the grey areas represent 95% predictor interval of the fitted line.

The observations in the RDA were further investigated by multiple linear regressions between photosynthetic parameters (F_v_/F_m_, chl *a* concentration, rETR_max_, E_k_ and α_PSII_) and the main explanatory variables (inorganic macronutrient concentrations, snow depth, ice thickness, brine salinity and DIC). Model outputs are summarized in Table 2. When controlling for phosphate and nitrite + nitrate concentration, only sampling depth significantly increased F_v_/F_m_ (p < 0.0001, Table 2). When plotted individually, F_v_/F_m_ was positively correlated with sampling depth (p < 0.0001) and accounted for 25.7% of the variability in F_v_/F_m_ (Fig 6a). After controlling for all three macronutrient concentrations, phosphate, nitrite + nitrate and sampling depth had a significant positive effect on chl *a* concentration, and accounted for 52% of the variability (Table 2). Silicic acid concentration did not have a significant effect on chl *a* concentration in the model (Table 2). Brine salinity, snow depth, phosphate and nitrite + nitrate concentration had a significant negative effect on rETR_max_ and α_PSII_, after controlling for the full model, which explained 60.1 and 52.4% of the variability, respectively (Table 2). When plotted individually, phosphate, nitrite + nitrate and silicic acid explained 16.0%, 17.5% and 3.5% of the variability in rETR_max_, respectively (Fig 4d–4f). In addition, ice thickness had a positive effect on α_PSII_, and silicic acid concentration had a significant positive effect on rETR_max_ and α_PSII_ (Table 1). E_k_ was significantly reduced by snow depth, ice thickness and phosphate concentration, when also controlling for brine salinity (Table 2). When plotted individually, E_k_ followed an exponential attenuation model of increased snow loading, explaining 42.6% of the variability in E_k_ (Fig 6b). Maximum rate of electron transport rate (rETR_max_) was negatively correlated with brine salinity (p < 0.0001, Fig 6c). Brine salinity accounted for 28.5% of the variability in rETR_max_. There was also a positive correlation between NPQ and brine salinity, explaining 15.1% of the variability in NPQ (p < 0.0001, Fig 6d).

**Table 2:** Variable estimator for the independent variables PO_4_ (μmol l^−1^), NO_2_ + NO_3_ (μmol l^−1^), Si (μmol l^−1^), sampling depth (m), snow depth (m), brine salinity (salinity units) and DIC concentration (μmol l^−1^). Independent variables were chosen based on Akaike Information Criterion, where NAs did not significantly contribute to the full model. Asterisk denotes statistical significance where p* ≤ 0.05, p** ≤ 0.01 and p*** ≤ 0.001.

	Fv/Fm	Log(Chl *a*)	Log(rETR_max_)	α_PSII_	E_k_
Intercept	0.213	1.26	3.97	0.283	164
Log(PO_4_)	0.0121	0.986***	−0.252***	−0.0562***	−16.6***
Log(NO_2_ + NO_3_)	−0.00565	0.525***	−0.126*	−0.0287**	NA
Log(Si)	NA	−0.215	0.205*	0.0682***	NA
Sampling depth	0.107***	0.574***	−0.163	0.0614***	−21.7**
Snow depth	NA	NA	−1.619***	−0.127*	−117***
Salinity	NA	NA	−0.0288***	−0.00597***	−0.900
DIC	NA	NA	NA	NA	NA
Adj. R^2^	0.408	0.520	0.601	0.524	0.476

## Discussion

In this study, we have investigated macronutrient variability and photophysiology of sea-ice algae in Antarctic pack ice, in a relatively under-studied sector of the Southern Ocean. Relationships between potential environmental drivers for the biomass distribution of sea-ice algae and bacteria have been examined, which are all well illustrated and summarized by the RDA. By using fluorescence kinetics as estimates of photosynthetic acclimation and performance in 237 sea-ice samples, we have identified snow depth, ice thickness and brine salinity to be strong environmental parameters affecting the PSII activity of sea-ice algae in the Amundsen and Ross Seas during summer. We also observed a strong positive relationship between microbial biomass, phosphate and nitrite + nitrate concentrations, which suggest high rates of remineralization and low photoautotrophic growth in samples with high amounts of biomass.

The sea ice in this study was sampled in an advanced melting stage. While the sea ice lacked the typical C-shaped salinity profile as in young and cold ice [15], the chl *a* maxima were most frequently located in the bottom section (55% of the cores), whereas the maxima in bacterial abundance were also often associated with the middle sections of the sea ice column. Chl *a* was increasing with depth in the sea ice column, which could partly be explained by low-light induced production of photosynthetic pigments, which has previously been described as a decrease of the ratio between particulate organic carbon and chl *a* as a response to snow accumulation [33]. Although brine-scaled chl *a* concentration reached relatively high levels (maximum 421 μg l^−1^), depth-integrated chl *a* concentrations in Southern Ocean sea ice have recently been estimated to be higher than in our study [18, 33]. We only focused on the brine community in the present study, and did not sample the surface-layer, which can contain significant amounts of biomass [18, 33]. Hence, disregarding the surface community may have resulted in an underestimate of biomass and productivity of the sea-ice microbial community in our study. When comparing to historical data during the past 25 years, our depth-integrated chl *a* concentrations are more comparable to spring and autumn concentrations, rather than summer values [18]. This could also indicate that the sea ice was sampled in an already late summer state, where significant amounts of algal biomass had already been flushed out of the ice as it was warming.

High concentration of chl *a* in sea ice is often accompanied by low nutrient concentrations, low DIC concentration, accumulation of dissolved organic matter, increased ammonia concentration, high bacterial abundance and increased rate of viral infections [1, 54–56]. In our study, high concentrations of chl *a* were also accompanied by high bacterial abundances, but neither pH nor DIC concentration correlated well with either chl *a* or bacterial concentrations. Also, unlike the studies cited above, chl *a* was positively correlated with macronutrient levels. The low degree of correlation between chl *a* and DIC suggests that these variables may not be good indicators for net DIC uptake or production during the time of sampling. However, DIC is also affected by other processes such as CO_2_ exchange and calcium carbonate dissolution or precipitation, which may have counteracted the DIC change due to primary production [1]. As the sea ice in this study was relatively warm and had a high brine volume (> 5%), the ice was generally considered permeable to fluids [17]. Therefore, the ice may have been flushed with seawater and thereby affected the accumulation of dissolved matter. It is also likely that the standing stock of algae was in a decaying post-bloom state during the time of sampling, and thereby physiologically less active, which was also supported by a negative correlation between chl *a* concentration and rETR_max_. This post-bloom situation is further supported by the high occurrence of potentially heterotrophic dinoflagellates in the sea ice identified in a concurrent study [34], that are often found grazing on declining phytoplankton blooms [57, 58].

The bulk concentrations of macronutrients were all in similar ranges as previously reported for Antarctic summer pack ice [2]. Concentrations of phosphate and nitrate + nitrite were generally enhanced near the bottom and top of the ice, due to replenishment of nutrient-rich under-ice water and surface flooding, respectively. Most of the interior ice was low in nitrate + nitrite as it was rapidly drawn down before the replenishment reached the middle sections of the ice. This pattern explains why the chl *a* maximum was generally located at the bottom of the ice, although it is not uncommon that high amounts of chl *a* is found in the middle sections of flooded pack ice [18]. There was a net drawdown of nitrate + nitrite and silicic acid in the sea ice compared to the TDL, supporting that the main primary producers were diatoms although a significant amount of the 18S rRNA sequences originated from dinoflagellates [34]. However, phosphate was both enriched and consumed, similar to what has previously been observed in Eastern Antarctica during wintertime [59]. This may suggest that there was an active recycling of phosphate in the sea-ice samples, which is also supported by the strong relationship between bacterial abundance and phosphate concentration.

It has previously been discussed that nutrient concentrations are the main controlling factor for ice algal biomass distribution in the Amundsen Sea in the late summer through surface flooding [33]. However, in that study the authors used snow loading as a proxy for nutrient replenishment, which is heavily confounded with light availability. Conversely we have assessed microalgal physiology by variable fluorescence, which is a good indicator for physiological stress in microalgae, including nutrient stress [36, 60]. Although bulk concentrations of nitrate + nitrite were relatively low in some parts of the ice, brine-corrected macronutrients were generally high in the present study, and correlated positively with chl *a* concentration and bacterial abundances. This may possibly be due to high rates of remineralization in this relatively warm summer sea ice [2]. Interestingly, rETR_max_ was reduced at high concentrations of phosphate and nitrite + nitrate, suggesting that the algae may experience stress and not being capable of maintaining their maximal potential photosynthetic rates.

Chl *a* was also positively correlated with inorganic macronutrient concentration, but did not correlate well with DIC concentration. In combination with high macronutrient concentrations in the brine, and little influence of high chl *a* concentration and bacterial abundance on the carbonate system, we believe that inorganic macronutrients are unlikely to have limited microalgal cellular physiology in this study. On the contrary, macronutrients (especially phosphate, but also nitrite + nitrate) were accumulated to high concentrations in the samples with high algal and bacterial biomass, suggesting an imbalance between production and consumption of nutrients. It is possible that a stressed and decaying standing stock of algae release large amounts of internal pools of compounds, a process to which the contribution of macronutrients levels in sea ice is still unknown. The release of nutrients from sea-ice algae during the melting process of sea-ice cores has previously been discussed in detail [2, 61]. Direct melts (i.e. no addition of filtered seawater) is often used to study macronutrient levels in sea ice, although this method implies osmotic stress for the microorganisms inhabiting the brine channels [2]. However, the osmotic shock during melting has not been shown to significantly impact bulk nutrient measurements in diatom-dominated sea-ice samples [62]. However, flagellate species are more susceptible to cell lysis during osmotic stress, but their general contribution to nutrient measurements in bulk ice is only believed to be minor [2]. Considering that dinoflagellates contributed to a significant fraction of the 18S rRNA sequences at these ice stations [34], it is possible that leakage during melt is higher than in the latter studies, and should be considered when interpreting these results. It is also possible that dinoflagellate grazing of the photoautotrophic community could release significant amounts of nutrients (e.g. ammonium) into the brine. However, grazing could not account for the reduced photosynthetic rates measured at high nutrient concentrations in this study. Microzooplankton grazing play an important role in planktonic ecosystems [63], but we currently know very little about grazing rates in sea ice and its impact on biogeochemical cycles.

Accumulation of phosphate and ammonium are common throughout the season in Antarctic pack ice, and can reach substantial values in the summer, probably due to the adsorption to organic particles [2]–a process that is still understudied in sea ice. It is possible that the high accumulation of extracellular polymeric substances (EPS), derived from sea-ice algae and bacteria, play an important role in sea-ice nutrient dynamics. It is therefore also possible that the high concentration of nitrite + nitrate in samples with high biomass is a result of high rates of nitrification of EPS-adsorbed ammonium [2]. As silicic acid does not adsorb to particles [2], EPS adsorption of phosphate and ammonium and a lower remineralization rate of silicic acid would explain how chl *a* concentrations predict phosphate and nitrite + nitrate levels well, but not silicic acid. Fucoxanthin concentration correlated well with chl *a* and did not provide a better fit of silicic acid over chl *a* concentration (6.1 vs. 5.6% of the variability), suggesting that diatoms may indeed have been the main primary producers in the samples.

Sea-ice micronutrient dynamics is still an under-studied field, but believed to play an important role in Southern Ocean nutrient cycling near the marginal ice zone [13]. Very little is known about the extent of micronutrient limitation in sea-ice algae, but it has previously been speculated that micronutrients could limit algal productivity under certain conditions in sea ice [13, 64]. Micronutrient limitation could potentially have caused a stagnation of photosynthetic rates in samples with high biomass and macronutrient concentrations in the present study, and could explain the observed PSII inactivity in samples with high biomass and high macronutrient concentration. However, this needs to be verified by concurrent measurements of micronutrient concentrations and uptake experiments.

In agreement with other studies [10, 65], bacterial abundance in sea ice followed a log-log relationship with chl *a* concentration, and were found in abundances anticipated for Antarctic summer sea ice [66]. Strong bacteria-algae associations have been reported in many other marine environments [67]. However, little is known about algae-bacteria interactions in sea ice. For instance, recycling of nutrients may be an important aspect for microalgal physiology. Bacterial assemblages accelerate the dissolution of silicic acid as they degrade the organic layer of the diatom frustules [68], and it was recently shown that sea-ice bacteria can recycle nitrogen through respiration of nitrogen-rich compatible solutes during downshifts of salinity [69]. Reduction of brine salinity can induce release and respiration of intracellular pools of nitrogen-containing compatible solutes [69], which could in turn contribute to ammonium levels. High accumulation of bacteria may provide an important feedback mechanism to the autotrophic community in sea ice, and could potentially explain the high brine-scaled macronutrient concentrations in some of our samples. This feedback mechanism may be especially important in sea ice going through melting stages with shifting brine salinities, as in the samples presented in this study. Hence, rapid regeneration of nutrients may partially explain the positive correlation of macronutrients and microbial biomass.

Pack-ice algal communities show a plastic response in PSII kinetics to elevated radiation, and can withstand relatively high irradiances [29]. As Southern Ocean pack-ice algal communities are distributed throughout the ice column, they experience a much greater range of PAR intensity than communities restricted to the bottom ice section [70]. Variability in snow loading also affects the light environment experienced in sea ice [33]. We have observed that E_k_ was strongly adjusted to the first ~0.2 m of snow loading, and subsequently leveled-out, following the exponential attenuation of PAR through matter. This observation supports the concept of a broad range of photoacclimation and plasticity in pack-ice algal communities [29], and suggests that the first 0.2 m of snow load may be critical for determining photoacclimation in sea ice. In addition, F_v_/F_m_ and α_PSII_ increased with sampling depth, highlighting the importance of efficient photoacclimation strategies at varying light intensities in sea ice. Plastic responses to radiation may be especially important for sea-ice algae in melting summer ice conditions in order to adjust their PSII performance to optimize energy demand at varying PAR levels. Plasticity may also be an important adaptation for the sea-ice community that is dispersed into the water column, as the ice is melting and thereby seeding the pelagic bloom [8].

Due to correlation between predictors in the multiple regressions models for rETR_max_ and α_PSII_, differentiating the effects of brine salinity and snow depth becomes challenging. Although the temperature gradient is less pronounced in surface-flooded summer pack ice, as compared to cold and growing sea ice [15], the microbial communities in this study were experiencing a broad range of brine salinity (5–50 units). A higher salinity resulted in decreased levels of the RLC-derived parameters rETR_max_ and α_PSII_, and increased NPQ. Interestingly, rETR_max_ and α_PSII_ were highest at the lowermost salinities, suggesting that this sea-ice algal community could be adapted to a lower salinity than experienced in the water column of the Southern Ocean [46]. We believe that it is unlikely that the high PSII capacity and performance at low salinity was an artifact from temperature differences in the sea ice. In laboratory experiments, sea-ice diatoms have previously been shown to increase F_v_/F_m_ by 6–9% when temperature was increased by 4°C [60, 71]. The latter temperature response is considerable smaller than for the temperature/salinity gradient in this study. Therefore, we argue that a potential temperature effect in the present study is negligible (−0.25 to −2.65°C), in relation to the wide salinity range (5 to 50 units) to generate the observed patterns in photosynthetic performance. Hence, we rather believe that radiation conditions and the broader range of salinity could directly affect the photophysiology of sea-ice algal communities. However, we cannot rule out the influence from the cofounding factors in the model.

A previous experimental study on algal assemblages from land fast sea ice suggests that decreases in salinity are more stressful than increases in terms of PSII performance [24]. This is contradictory to our study, where acclimation to lower salinity resulted in increased photosynthetic performance. The differences between the studies may be due to species-specific responses in pack ice vs. fast ice. For instance, the physical characters of land fast ice differ considerably compared to pack ice, and generally contain different algal and bacterial communities [19, 34]. The properties of the ice may have selected for a community more tolerant to the lower range of sea-ice brine salinity. This may also be promoted by the accumulation of biomass in the surface layer in the snow-ice intersect [33, 38]. As pack ice is often surface-flooded due to high accumulation of snow, top-infiltration of water containing both surface-layer algae and nutrients is likely to reach the interior ice if the slush goes through freeze-thaw cycles [72]. When the slush freezes, brine is formed that can generate convection in the ice if the brine salinity is greater than in the interior ice, and thereby replenishing the ice with both nutrients and microorganisms. Such convection is more likely to occur in warm ice where the brine salinity is low, like in the present study. The surface communities have been described to hold a high photosynthetic capacity [33, 38], and are most likely acclimated to low salinities. Hence, the infiltration of surface communities into the interior ice could also explain the patterns in our data, and could have important consequences for primary production in Antarctic sea ice.

As the areal extent and thickness of sea ice in the Amundsen Sea has diminished during the last 35 years [27], the physical characters of sea ice are also affected (e.g. thinner ice, more melt ponds and rotten ice), which could have consequences for sea-ice microbial ecosystems. In the Arctic, drastic changes in sea-ice thickness have resulted in extensive under ice blooms of phytoplankton [73], that have significant consequences for benthic ecosystems and biogeochemistry [74]. Ice features associated with salinity and radiation extremes, such as freshwater melt ponds, under-ice melt lenses and rotten ice, will most likely become more common as the melt season is getting longer due to global warming, and will most likely select for microalgal species capable of acclimating to these extremes. Future changes in sea ice thickness and snow precipitation may also promote nutrient fluxes in the sea ice via surface flooding, which may in turn enhance algal and bacterial production in the brine channels. This may be particularly important during the late summer, when the autotrophic community becomes stagnant due to nutrient depletion, and in sea ice in areas that currently have low amounts of snow accumulation.

As the sea-ice situation in polar oceans is currently undergoing rapid changes, it is important to understand how physicochemical properties and nutrient distribution affect key ecosystems and biogeochemical processes. There is also relatively little physiological and biogeochemical data from pack ice in the Amundsen Sea available, which is a key area for studying diminishing sea-ice situation in the Southern Ocean. Although few studies that focus on sea-ice microalgal photophysiology are as extensive (both geographically and vertically) as the present study, results from this work are fairly consistent with previous findings [2, 10, 29, 33, 75]. We have reported a plastic response in photophysiology that highlights the capacity for sea-ice algae to acclimate to different light environments. We observed a strong relationship between chl *a* and phosphate and nitrite + nitrate, suggesting that the autotrophic community was in a decaying state during sampling, where microbial remineralization exceeded algal uptake. Although macronutrient concentrations did not seem to be limiting photosynthetic rates in the brine channels of surface-flooded pack ice in our study, we speculate that sea ice in advanced melt stage may lack certain micronutrients to sustain primary production in sea ice similar as to the Southern Ocean overall.

## Supporting information

S1 Fig Light saturation point of photosynthesis (E_k_) of sea-ice algae was positively correlated (p < 0.02, Pearson’s correlation) with time after the sampling the sea-ice cores in two of the eleven stations. The numbers 4–15 represent station number during the OSO10/11 cruise in the Amundsen and Ross Seas.
(EPS)

S2 Fig Incoming surface photosynthetic active radiation (PAR) irradiance during the OSO10/11 cruise in the Amundsen and Ross Seas. Data were collected using a 2π PAR sensor placed on top of the bridge of *IB Oden*.
(EPS)

S3 Fig Relationships between dissolved inorganic carbon (DIC) and (a) chl *a* concentration and (b) bacterial abundance in summer sea ice during the OSO10/11 cruise. The grey area in (**a**) represents 95% prediction intervals of the fitted line. All concentrations are scaled to brine volume.
(EPS)
